# Anesthetic management for cytoreductive surgery of pseudomyxoma peritonei with high intra-abdominal pressure: A case report

**DOI:** 10.3389/fsurg.2022.1017500

**Published:** 2023-01-16

**Authors:** Yan-Jun Lin, Liang-Yuan Lu, De-Qiang Tao, Zhan-Min Yang

**Affiliations:** Department of Anesthesiology, Aerospace Center Hospital, Beijing, China

**Keywords:** pseudomyxoma peritonei, cytoreductive surgery, hyperthermic intraperitoneal chemotherapy, intra-Abdominal hypertension, anesthetic management

## Abstract

Anesthetic management for patients of pseudomyxoma peritonei (PMP) is challenging. This case report describes a patient of PMP with high intra-abdominal pressure. Intubation was performed in lateral position; the intraabdominal pressure was relieved slowly to prevent significant hemodynamic changes. Additionally, positive pressure ventilation was performed to reduce the risk of re-expansion pulmonary edema. During the operation, transfusion and infusion therapy was performed with target-mediated fluid therapy according to stroke volume variation (SVV) and cardiac index (CI) and blood gas analysis.

## Introduction

Pseudomyxoma peritonei (PMP) is a rare clinical syndrome. Cytoreductive surgery (CRS) combined with hyperthermic intraperitoneal chemotherapy (HIPEC) is a safe and effective treatment for PMP (1). The surface of the peritoneal membrane and the intraperitoneal organs in patients with abdominal mucosa are common sites of tumor metastasis and dissemination, and distant metastasis is very rare. With the growth of the tumor, the intraabdominal pressure will increase in a period of time. And the edema in lower limb is a result of compression of the inferior vena cava. Besides, the CRS combined with HIPEC treatment can last from 4 h to more than 12 h. The postoperative period always requires an extended hospital stay with many reported complications including bleeding, sepsis, anastomotic leak, and renal failure. Therefore, perioperative anesthetic management of CRS patients with HIPEC can be very challenging. We herein describe a PMP patient with high intraabdominal pressure and analyze this case from the perspective of anesthetic management.

## Case presentation

### Patient information

**Figure 1:**
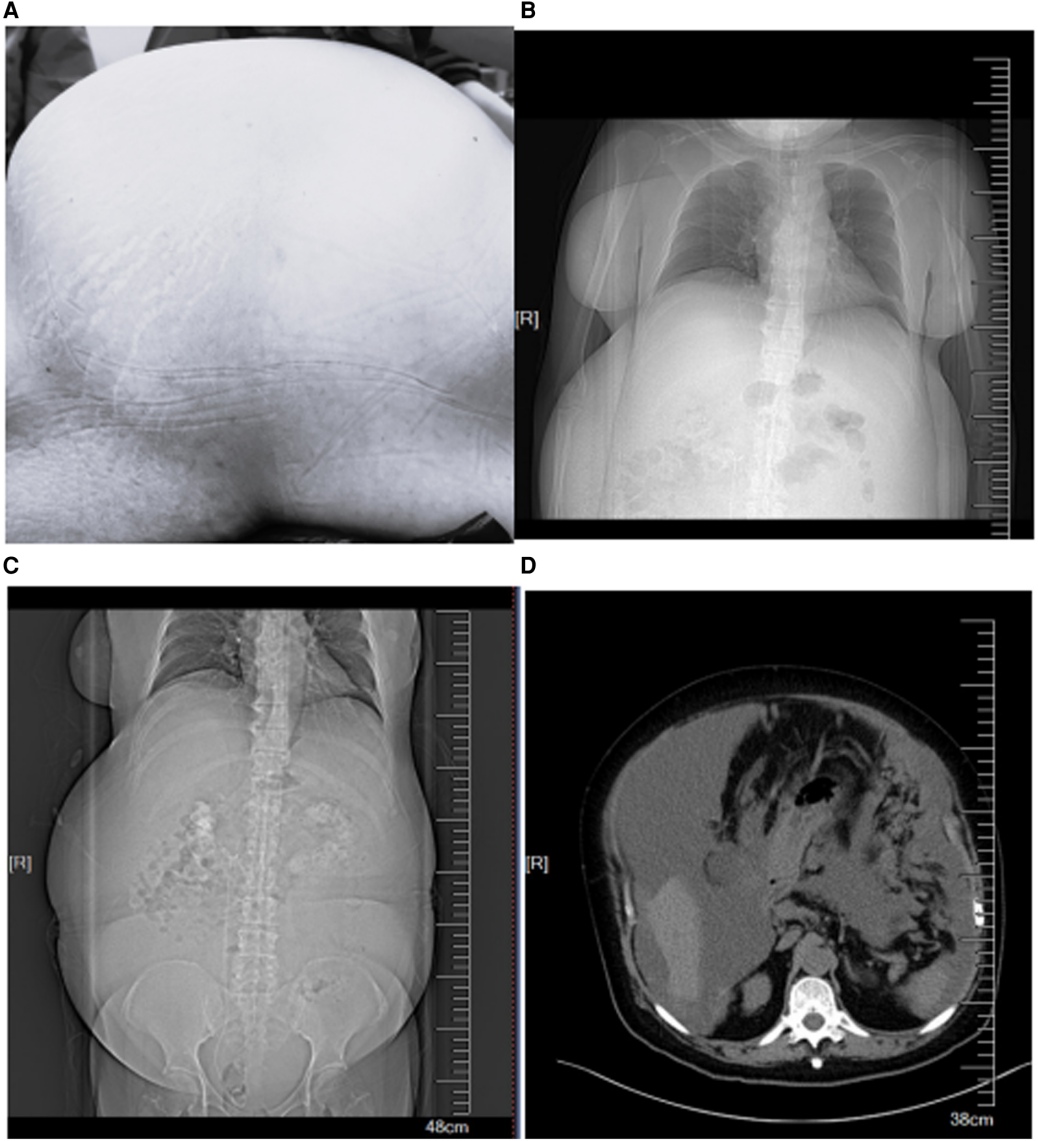
(**A**) The increased abdominal circumference. (**B**) Chest computed tomography revealed an elevated diaphragm. (**C,D**) Abdominal computed tomography revealed multiple cystic solid mass and nodular soft tissue density.

A 62-year-old woman (height, 161 cm; weight, 88 kg) was admitted to the hospital because of a 3-month history of increased abdominal circumference, shortness of breath and disable of lay down. The patient was diagnosed as PMP by pathology of ascites. We have got the patient's written informed consent to publish the case report. The patient had a history of hypertension for 11 years, paroxysmal atrial fibrillation for about one month. The blood pressure was controlled by nifedipine. Before the operation, the patient feels dyspnea and must have the passive position of left lateral and reverse Trendelenburg. The abdominal circumference was high to 115 cm (Figure 1A). Chest computed tomography revealed an elevated diaphragm and lungs were compressed (Figure 1B), and multiple cystic solid mass and nodular soft tissue density shadows were seen in abdominal cavity and pelvic cavity, with unclear boundaries and internal separations (Figures 1C,D). The left ventricular diastolic function was decreased, and the left ventricular ejection fraction was 58%. Laboratory tests showed renal insufficiency and coagulation disorders because of anticoagulant therapy. Arterial blood gas analysis showed the following: pH: 7.415, PaCO_2_: 31.1 mmHg, PaO_2_: 83.6 mmHg, lactate: 1.74 mmol/L, potassium: 4.74 mmol/L, sodium: 132 mmol/L, calcium: 1.14 mmol/L, FiO_2_: 21%, and SpO_2_: 96.7%.

### Anesthesia

The patient was in the passive position of left lateral and reverse Trendelenburg when she was in the operation room. The patient's electrocardiogram, blood pressure, SpO_2_, and bispectral index were monitored. A radial artery catheter was placed, intra-arterial blood pressure (IBP), CI, and SVV by using FloTrac vigileo system were monitored. Arterial blood pressure was 148/82 mmHg, heart rate was 97 beats /minute, and SpO_2_ was 96%. Arterial blood gas analysis showed the following: pH: 7.34, PaCO_2_: 34 mmHg, PaO_2_: 80mmHg, lactate: 0.9 mmol/L, potassium: 4.6 mmol/L, sodium: 138 mmol/L, calcium: 1.14 mmol/L, hemoglobin (Hb) 9.6 g/dl, FiO_2_: 21%, and SpO_2_: 94.9%. Before anesthesia, 40 μg of dexmedetomidine was infused within 10 min and gastric contents were suction through a gastric tube. The patient was preoxygenated with 100% oxygen at 6 L/minute for 5–10 min. Anesthesia induction was performed with 20 mg of etomidate, 20 μg of sufentanil, and 50 mg of rocuronium. A 7.0 endotracheal tube was inserted with the help of a video assisted device at the first position, and ventilation was performed. The tidal volume was set at 6 to 8 ml/kg, the respiratory rate was 13 to 15 breaths/minute, and the inhalation: expiration ratio was 1:2. Propofol, remifentanil, and sevoflurane were used to maintain anesthesia. Maintenance anesthesia was performed by static inhalation compound anesthesia with propofol 4–8 mg/kg/h, remifentanil 4–8 ug/kg/h, and inhaled sevoflurane 1%–2%, and the depth of anesthesia was adjusted according to the BIS value (bispectral index) to maintain the BIS at 40–60. After the intubation, the patient was slowly put in the supine position and there were no significant changes in blood pressure and heart rate. Then a right internal jugular venous catheter was placed guided by ultrasound and the central venous pressure and oropharyngeal temperature was monitored.

### Intra-operative

**Figure 2:**
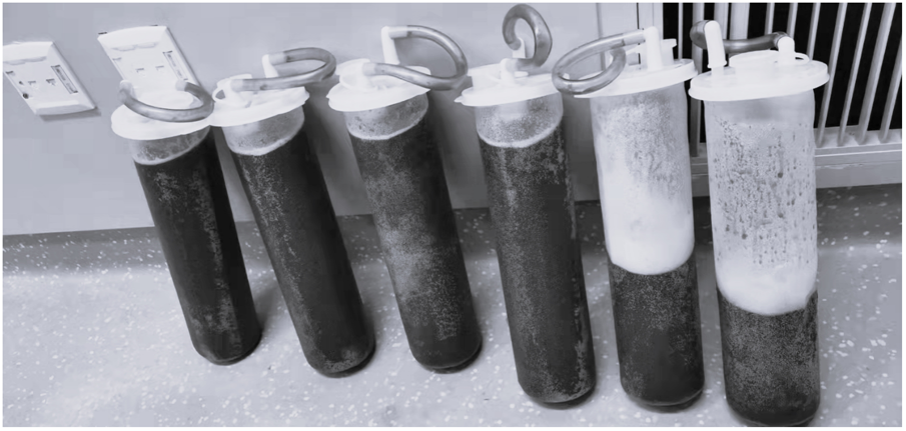
The ascitic fluid of the patient.

The surgeon created a small incision for paracentesis. 11,000 ml of ascitic fluid was drained in 30 min (Figure 2). In this period, 800 ml of Lactated Ringer's solution was administered. The airway pressure decreased from 30 cmH_2_O at the beginning of the operation to 21 cmH_2_O, the CI increased from 1.8 L/min^.^m^2^to 2.2 L/min^.^m^2^, SVV decreased from 13 to 2, central venous pressure (CVP) decreased from 23.3 cm H_2_O to 15.7 cmH_2_O. There were no significant changes in blood pressure and heart rate. After ascites drainage, we performed blood gas analysis before the start of the main steps of CRS. Arterial blood gas analysis showed the following: pH: 7.25, PaCO_2_: 42 mmHg, PaO_2_: 205 mmHg, lactate: 0.9 mmol/L, potassium: 4.8 mmol/L, sodium: 135 mmol/L, calcium: 1.18 mmol/L, Hb 8.2 g/dl, bases excess (BE) −7.8,FiO_2_: 60%, and SpO_2_: 97.0%. The respiratory rate was adjusted to 10 to 12 breaths/minute, the positive end-expiratory pressure was set at 5 cmH_2_O, FiO_2_ was adjusted from 1.0 to 0.4.

**Figure 3:**
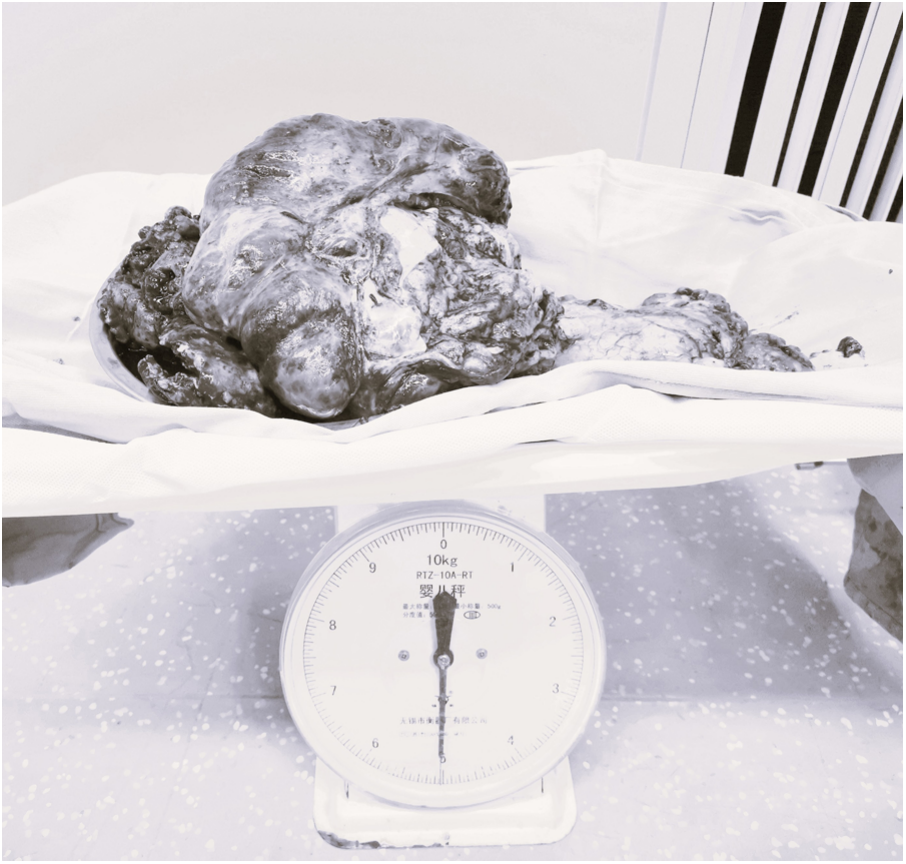
The diseased or metastasized tissues.

CRS was continued for about 3.5 h and about 5 kg of diseased or metastasized tissues were removed (Figure 3). During the surgery, the patient experienced 3000 ml of blood loss and excreted 600 ml of urine, was administered 3800 ml of lactated Ringer's solution and 2500 ml of colloidal solution and 200 ml 20% albumin. During the CRS, intermittent arterial blood gas analysis was performed. When the hemoglobin was decreased from 9.6 g/dl to 6.0 g/dl, then 7 units of suspended red blood cells and 1,000 ml of fresh frozen plasma were given. 100 ml of sodium bicarbonate were given when BE was decreased to −7.8 according to the blood gas analysis. Before HIPEC, the oropharyngeal temperature was deceased from 36.4°C to 35.2°C in spite of the usage of heating blanket. The detail of blood gas analysis at different time points during the surgery was shown in Table 1.

**Table 1:** Blood gas analysis at different periods during operation time.

Time points	Bleeding volume (ml)	pH	PaCO_2_ (mmHg)	PaO_2_ (mmHg)	Lactate (mmol/L)	Potassium (mmol/L)	Sodium (mmol/L)	Calcium (mmol/L)	Hb (g/dL)	FiO_2_ (%)	SpO_2_ (%)
Before anesthesia		7.34	34	80	0.9	4.6	138	1.14	9.6	21	94.9
Starting of ascites drainage		7.25	42	205	0.9	4.8	135	1.18	8.2	60.0	97.0
1.5 h after ascites drainage	2,000	7.33	44	167	0.6	4.8	137	1.11	6.0	40.0	95.6
2.5 h after ascites drainage	1,000	7.35	37	192	0.8	4.8	137	1.05	6.5	40.0	96.0
4 h after ascites drainage		7.32	39	189	0.8	4.6	139	0.97	7.5	40.0	96.4
Ending of surgery		7.32	40	184	0.6	4.2	142	1.11	8.5	40.0	95.6

Note: Hb, hemoglobin concentration.

### Postoperation

HIPEC was performed after CRS in a closed-loop manner: after the latex drainage tubes were put in the pelvic and bilateral upper abdomen, the skin was closed and the latex drainage tubes were fixed by continuous suture. The circulation was controlled at the speed of 800 ∼1,000 ml/min. The temperature was set to 41 °C. Mitomycin (MMC) 30 mg and Cisplatin (DDP) 60 mg were dissolved in 4,000 ml of normal saline. HIPEC was continued for 60 min. In this period, the oropharyngeal temperature was increased to 37°C. Torsemide 5 mg was given during this period and another 800 ml of urine was excreted.

**Figure 4:**
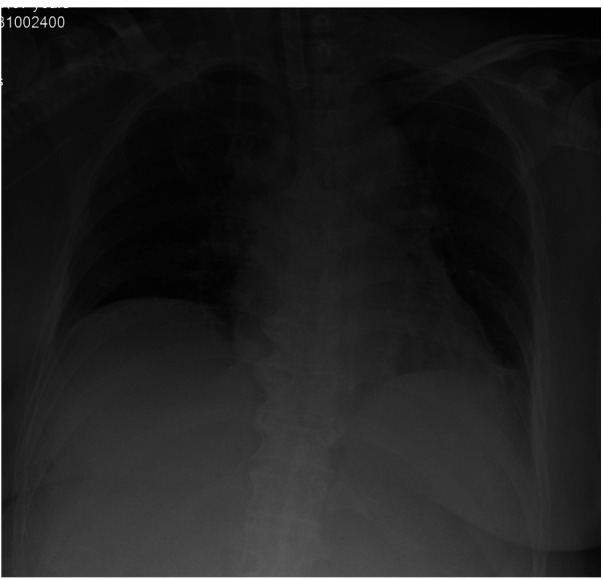
Chest x-ray revealed a normal position of diaphragm.

In the whole period of surgery, the hemodynamic was maintained by noradrenaline when needed. IBP and HR were controlled at 90–110/40–50 mmHg and 60–75 beats/minute. CI and SVV were maintained at 1.9–2.6 L/min.m^2^and 5–16. Lac was decreased from 0.9 mmol/L at the beginning to 0.6 mmol/L at the end of the surgery. Hb was decreased from 9.6 g/dl at the beginning to 8.5 g/dl at the end of the surgery.The patient was transferred to intensive care unit (ICU) after the operation. x-Ray of Chest revealed the elevated diaphragm was descended at a normal position (Figure 4). Patient underwent five separate sessions of intraperitoneal thermoperfusion chemotherapy from the 3rd to the 8th day after surgery; She had her abdominal drain removed on the 11th postoperative day, and was discharged on the 15th postoperative day. No other complications were observed.

## Discussion

In this patient, the abdominal circumference was high to 115 cm, the high intra-abdominal hypertension induced dyspnea and edema of both lower limbs. The large amount of ascites lifted the diaphragm, reducing the functional residual volume, and shortening a hypoxia safety time of apnea-hypoxemia time (AHT). These increase the risk of aspiration and rapid desoxysaturation during anesthesia induction, so the airway was carefully evaluated before surgery and prepared for a difficult airway if necessary. To prevent the chance of aspiration, we performed full attraction to the gastric contents before the anesthesia induction. Because of the passive position, we performed the anesthesia induction and intubation at this position after 5–10 min of preoxygen. The patient was slowly put in the supine position and the hemodynamics changes were monitored for avoiding the rapid decrease in return blood volume caused by the huge abdominal pressure compressing the inferior vena cava after rapid supine position, and the decrease will result in great hemodynamic fluctuations. In the operation, we created a small incision for paracentesis to avoid a significant hemodynamic change due to the rapid decrease of intraabdominal pressure and it was proved effective in this patient.

Low intravascular volume is very common during CRS due to ascites drainage and blood loss. A large amount of fluid transfusion will be needed. The study of Schmidt showed that all patients were supplemented with 4900m1on average of crystal fluid and 2,500 ml on average of colloidal fluid. Treatment with open fluid to supplement the intraoperative loss of body fluids is at risk of volume overload, tissue edema, and severe abdominal complications (2). However, restrictive fluid therapy also has a risk of hemodynamic instability, tissue hypoperfusion, organ damage, and increased nephrotoxicity of chemotherapeutic agents. Goal-directed fluid therapy (GDFT) can realize individualized treatment which will reduce the incidence of intestinal and systemic complications and improve prognosis in these patients (3, 4). After ascites drainage, SVV decreased from 13 to 2. We speculated that this was because this patient had about 3 months of ascites formation, and most of the intraoperative ascites drainage did not participate in the effective circulating volume. However, because of the increase in abdominal pressure caused by the large amount of ascites, it may have caused an obstruction in the return of effective circulating volume to the lower extremities, which was caused by the increase in effective circulating volume after the abdominal pressure was relieved. Approximately 1/3–1/2 of these patients needed concentrated erythrocytes and fresh frozen plasma. 7 units of suspended red blood cells and 1000 ml of fresh frozen plasma were given in this patient according to the blood loss and hemoglobin of blood gas analysis.

High intra-abdominal pressure (IAP) at the level of 18–22 mmHg during HIPEC is feasible and safe in patients diagnosed with PMP (5). But the body temperature will rise to (38.3 ± 0.97)°C despite a low body temperature due to the long operation. In this patient, heating blanket and blood transfusion and infusion heating instrument were used before HIPEC and were stopped 30 min before HIPEC coupled with lower temperature of the room to avoid a high body temperature (>41°C). High loss of fluid and dilatation of peripheral blood vessels may lead to insufficient renal perfusion and oliguria or even anuria in these patients. Furthermore, chemotherapeutic agents like mitomycin C and cisplatin have significant nephrotoxicity, the treatment of appropriate amount of fluid infusion and low dose of diuretic is important. We often use 5–10 mg of Torsemide during HIPEC. With the growth of the tumor in PMP patients, abdominal pressure will increase in a long term which will reduce the reflux of the inferior vena cava and cause lower limb edema; after the removal of abdominal mucus,the abdominal pressure may decrease dramatically which may cause a significant change of the blood volume. We made a small incision for paracentesis slowly to avoid a dramatically hemodynamic change. In this case, we did not observe the changes in abdominal pressure values before and after the drainage of ascites, which is something we need to improve and refine when we encounter similar cases in the future.

Cytoreductive surgery combined with hyperthermic intraperitoneal chemotherapy operation is complicated, with a huge incision, completely opened peritoneal cavity, and large fluid loss, especially in HIPEC stage, which can induce significant changes in hemodynamics, high temperature and coagulation dysfunction (6). Anesthesiologists must be familiar with the pathophysiological changes of CRS-HIPEC to maintain the patient's hemodynamicthe and body temperature and coagulation function in the normal or close to the normal paradigm.

## Data Availability

The original contributions presented in the study are included in the article/Supplementary Material, further inquiries can be directed to the corresponding author/s.
